# Phytochemistry, Antimicrobial, Analgesic, Antibiofilm, Diuretic Activities, and Acute Toxicity of Bark Extracts From Three Plants (Khaya senegalensis, Ocimum americanum, and Cassytha filiformis) Collected in Benin

**DOI:** 10.1155/tswj/1106284

**Published:** 2026-02-24

**Authors:** Funkè F. Assouma, Atchadé Pascal Tchogou, Cyrille A. Vodounon, Gautier Roko, Machioud Maxime Sangaré, Durand Dah-Nouvlessounon, Bassitath Bello, Bawa Boya, Rachidatou Karimou, Basile Konmy, Adolphe Adjanohoun, Lamine Baba-Moussa, Haziz Sina

**Affiliations:** ^1^ Laboratory of Biology and Molecular Typing in Microbiology, Department of Biochemistry and Cell Biology, Faculty of Science and Technology, University of Abomey-Calavi, Abomey-Calavi, Benin, uac.bj; ^2^ Experimental and Clinical Biology Unit (UBEC), Medical and Pharmaceutical Biotechnology Research Laboratory (LaBiMeP) of the National School of Applied Biosciences and Biotechnology of Dassa-Zoumé (ENSBBA), National University of Sciences, Technologies, Engineering, and Mathematics (UNSTIM), Dassa-Zoumé, Benin; ^3^ Laboratory of Natural Sciences and Applications, 72 ENS-UNSTIM, Natitngou, Benin; ^4^ Department of Animal Physiology, Faculty of Science and Technology, University of Abomey-Calavi, Cotonou, Benin, uac.bj; ^5^ National Agronomic Research Institute of Benin, Cotonou, Benin

**Keywords:** acute toxicity, Benin, biological activities, medicinal plants, secondary metabolites, urinary infections

## Abstract

In Beninese traditional medicine, *Khaya senegalensis*, *Ocimum americanum*, and *Cassytha filiformis* are commonly used to treat urinary tract infections (UTIs). This study aimed to evaluate their phytochemical composition, antimicrobial and anti‐biofilm activities, analgesic and diuretic effects, and acute toxicity. Qualitative phytochemical screening was performed using staining and precipitation methods. Plant materials were extracted with 70% ethanol, yielding a hydroethanolic extract. Antibacterial activity against multidrug‐resistant uropathogenic strains was assessed using disk diffusion and broth microdilution assays. Anti‐biofilm effects were evaluated with a colorimetric method. Analgesic and diuretic activities were tested in vivo in Wistar rats. Acute toxicity of hydroethanolic extracts was assessed over 14 days following OECD guidelines. Phytochemical analysis revealed alkaloids, flavonoids, glycosides, saponosides, tannins, reducing compounds, and mucilage in various plant parts. All extracts exhibited antibacterial activity against Staphylococci and uropathogenic Enterobacteria, with inhibition zones of 13.8–21.2 mm and MICs of 3.5–9 mg/mL. Both aqueous and hydroethanolic extracts reduced bacterial biofilm formation to varying degrees. At 200 mg/kg, the hydroethanolic extracts significantly reduced acetic acid–induced pain. *K. senegalensis* and *C. filiformis* exhibited diuretic activity comparable to furosemide. No toxic effects were observed at 2000 mg/kg. The antimicrobial, anti‐biofilm, analgesic, and diuretic properties of these plants support their use in managing UTIs. Their favorable acute toxicity profile further highlights their therapeutic potential and supports the development of improved traditional medicines.

## 1. Introduction

Urinary tract infections (UTIs) occur when microorganisms invade the urinary tract, leading to inflammation and various clinical symptoms [1]. UTIs are prevalent and affect 27.6% of people of all ages and genders [2]. However, the emergence of multiresistant and virulent bacteria has made treating UTIs challenging and a significant public health concern [3].

Increased antibiotic resistance among germs causing UTIs has recently been observed [4–6]. This global issue significantly impacts human health, animal health, the environment, and the economy, as pointed out by Tang et al. [7]. Additionally, bacteria’s ability to form biofilm has been linked to antibiotic therapy failure [8], resulting in chronic and recurrent infections that are challenging to eradicate [9]. Analgesics to relieve pain and diuretics to eliminate germs through urination are also necessary to treat UTIs.

Given the resistance and virulence of uropathogenic bacteria, developing new strategies to manage UTIs and overcome antibiotic resistance is crucial. Medicinal plants offer a natural alternative to synthetic or semi‐synthetic chemicals and contain biologically active substances with antimicrobial, anti‐biofilm, analgesic, and diuretic properties [10]. Traditional medicine, especially in Africa, highly values and widely utilizes plants to treat and prevent diseases, including bacterial infections [11]. Cranberries and Chinese herbs have been proven effective in preventing and treating UTIs due to their antimicrobial properties [12]. In contrast, other plants with similar properties can help combat antibiotic resistance in uropathogenic strains [13].

However, despite the widespread use of medicinal plants, there is a lack of information regarding their proper use and valid biological properties. Furthermore, herbal medicines are often mistakenly considered to have fewer adverse effects than Western/allopathic medicines [14]. Therefore, improving the recipes traditionally used to treat or prevent diseases through phytochemical studies and evaluating medicinal plants’ biological activities and toxicities is necessary [15]. The present study aims to investigate five medicinal plants used in managing UTIs in traditional medicine in Benin to determine their physicochemical parameters, antibacterial, anti‐biofilm, analgesic, and diuretic activities, as well as the toxicity of their extracts.

## 2. Materials and Methods

### 2.1. Plant Collection and Pulverization

The study involved the collection and identification of medicinal plants in southern Benin, namely *Ocimum americanum* Sims (stem bark), *Cassytha filiformis* L. (whole aerial parts), and *Khaya senegalensis* (Desr.) A. Juss. (stem bark). These species were selected because they were the most frequently cited in the ethnobotanical survey conducted by Assouma et al. [16] on medicinal plants used in the treatment of UTIs in Benin.

Plant identification was carried out at the National Herbarium of Benin, where voucher specimens were deposited under the following numbers: *O. americanum* (voucher no. YH 753/HNB), *C. filiformis* (voucher no. YH 750/HNB), and *K. senegalensis* (voucher no. YH 752/HNB).

After collection, the plant organs used in this study (stem bark of *O. americanum* and *K. senegalensis*, and whole aerial parts of *C. filiformis*) were washed with clean water to remove extraneous materials. The samples were shade‐dried in a well‐ventilated room at 25°C ± 2°C until constant weight was achieved (approximately 14 days). The dried materials were then ground into a fine powder using an electric grinder and stored in sealed sachets until further analysis.

### 2.2. Plant Extraction

Two methods were utilized to extract the plant material: aqueous and ethanolic. For the aqueous method, 50 g of plant powder was soaked in 500 mL of distilled water for 72 h, then filtered twice through absorbent cotton and once through Whatman No. 1 paper. The filtrate was dried at 50°C to produce the total aqueous extract. The hydroethanolic method involved immersing 50 g of plant powder in 500 mL of 70% ethanol for 72 h, then filtering twice through absorbent cotton and once through Whatman No. 1 paper. The filtrate was concentrated in a Rotavapor under vacuum at 50°C, then dried and kept in sterile glass bottles at 4°C.

The extraction yield was calculated using the formula R (*%*) = Me/Mv × 100, where R (%) is the yield percentage, “Me” is the mass of dry extract, and “Mv” is the mass of plant material used.

### 2.3. Phytochemical Screening

The phytochemical screening was performed on the powdered plant using qualitative tests [17]. This involved analyzing the different reactions of coloring or precipitation to determine whether various secondary metabolites were present or absent in the plant.

### 2.4. Biological Activities of Plant Extracts

To determine the plant’s biological activities, we tested it against uropathogenic bacterial strains resistant to multiple antibiotics, produced biofilm, and possessed several virulence genes. These bacterial strains included *Staphylococcus* spp. and *Enterobacteriaceae*, previously isolated by Assouma et al. [5, 6].

#### 2.4.1. Antimicrobial Activity

Assessing the effectiveness of antimicrobial activity involves conducting a sensitivity test of the extracts on the strains. Then, determining the minimum inhibitory concentrations (MIC) and minimum bactericidal concentrations (MBC) are the antibacterial parameters analyzed.

To test sensitivity, small discs were created from Whatman No. 5 filter paper (*θ* = 6 mm). The discs were sterilized to prevent contamination during the experiment (120°C for 15 min). A preculture of bacteria (one colony in 10 mL of nutrient broth incubated for 18 h) was diluted in sterile distilled water and spread on the surface of Petri dishes filled with MH agar medium [18]. This solution was then spread on Petri dishes containing MH agar medium. The discs were placed on each dish’s surface and soaked in aqueous and hydroethanolic extracts at 20 mg/mL. DMSO was used as a negative control. The inhibition diameters were measured using a graduated ruler after incubating the Petri dishes at 37°C for 24 h.

The macro‐dilution method in a liquid medium was used to determine the MIC [18]. We labeled 10 test tubes from T1 to T10 and added 100 *μ*L of sterile distilled water to all tubes except T1. We then introduced 1 mL of the extract at the 20 mg/mL starting concentration into tubes T1 and T2. From tube T2, we carried out successive dilutions of 1/2 reason until tube T9. We added 100 *μ*L of inoculum at 10^6^ CFU/mL of MH nutrient broth to all tubes, resulting in a final volume of 2 mL. The tubes were incubated at 37°C, and after 24 h, we examined the turbidity of the tubes and compared them with the control tube T10.

We followed the method to determine the MBC [19]. First, we determined the MIC and then inoculated all the tubes starting from the MIC towards higher concentrations on Petri dishes containing MH agar medium using a platinum loop. The dishes were then incubated in an oven at 37°C for 24 h. After observation, the extract concentration that showed no microbial growth was deemed the MBC.

We also used the CMB/CMI ratio to determine the antimicrobial effect of the extracts. The extract was considered bactericidal if the ratio was less than or equal to 4. The extract was considered bacteriostatic if it was more significant than 4 [20].

#### 2.4.2. Anti‐Biofilm Formation Activity

A 96‐well flat‐bottomed polystyrene plate was used following the method outlined by Nikolić et al. [21] with some alterations to test the extracts’ effectiveness in preventing biofilm formation. Each well was filled with 100 *μ*L of MH broth containing 10^8^ CFU of each microbial strain. Then, 100 *μ*L of extract at a concentration of 20 mg/mL was added to each well, while wells without extract served as controls. All plates were incubated at 37°C for 48 h, after which the supernatant was removed, and any floating cells were eliminated by washing the wells with sterile distilled water. The plates were air‐dried for 30 min, then stained with 200 *μ*L of a 0.1% aqueous solution of crystal violet. The plates were left at room temperature for 15 min to observe any biofilms formed. After incubation, the excess dye was removed by washing the plate three times with sterile distilled water. The microbial cell‐bound dye was solubilized by adding 95% ethanol to each well, and after incubation for 15 min, the optical density was measured by a spectrophotometer at 570 nm [22]. Using the formula: *P* *B*I = (OD (control) − OD (test))/(OD (control)) × 100, the percent biofilm inhibition (PBI) was calculated.

### 2.5. Evaluation of the Analgesic Activity of the Extracts

The analgesic activity of the extracts was tested using the writhing test. This method involves inducing pain in rats by injecting them with acetic acid (3%) via the intraperitoneal (IP) route. This injection causes the rats to experience abdominal cramps, which are detected by observing contortioned movements of the dorsal‐abdominal musculature. The analgesic effect is evaluated by counting the number of cramps within 30 min [23].

Thirty minutes after administering various treatments, all rats were injected intraperitoneally with 0.1 mL of a 3% acetic acid ion solution. The number of writing (NC) for each rat was counted for 30 min. The percentage of cramp inhibition (PCI) was calculated using the formula: PCI = (NCC − NCT)/(NCC) × 100, where NCC is the average number of contortions at the control group level and NCT is the average number of distortions at the level of the treated groups.

### 2.6. Evaluation of the Diuretic Effect of Extracts

To determine the diuretic effect of plants, we followed the method outlined in Rahman et al.’s work [24]. We divided 21 rats into seven groups of three rats each. The first group, which served as a negative control, received saline (10 mL/kg, p.o). The second group received furosemide (10 mg/kg, p.o) in the vehicle (NaCl 0.9%), while the remaining groups were given extracts at doses of 200 mg/kg body weight. After treatment, we hydrated all rats with saline (25 mL/kg) and placed them in metabolic cages to separate urine and feces. We collected the urine over 6 h and measured the volume using a graduated cylinder. Using an analyzer, we analyzed the electrolyte composition (Na+, K+, and Cl−) of the urine collected from each group. We did not allow the animals to eat or drink throughout this process. To calculate the volumetric urinary excretion (EUV), we used the following formula: EUV = (Volume excreted)/(Volume administered) × 100.

### 2.7. Acute Toxicity

To determine the acute toxicity of our extracts, we followed the OECD 407 guidelines over 14 days [25]. Based on weight, we randomly assigned mixed‐sex experimental animals into six groups consisting of three animals each. Five groups were given a daily oral dose of our extracts at 2000 mg/kg body weight, while one group served as the control (rats were fed without extract).

Observation was conducted every 30 min for 8 h on the first day and once daily for the next 13 days. We observed the animals for symptomatologic disorders such as agitation, lack of appetite, eye color, motor difficulties, diarrhea, lethargy, and dyspnea. Blood samples were collected from each rat by puncturing the retro‐orbital sinus before and 14 days after treatment using EDTA and ordinary tubes for biochemical and hematological analyses. In addition, at the end of treatment, one rat per batch was sacrificed, and liver and kidney samples were collected in clean bottles for histological tests.

### 2.8. Data Processing and Statistical Analysis

We used SPSS software version 26.0 (IBM SPSS, Chicago, IL, United States) and GraphPad Prism software version 9.0.2 (GraphPad Software, United States) to analyze the data on physical activities and toxicity. We conducted the Shapiro–Wilk normality test and compared the data that did not show a normal distribution of variables using the Kruskal–Wallis nonparametric test followed by Dunn’s test. For data with a normal distribution of variables, we used a one‐way analysis of variance (ANOVA) followed by Dunnett’s post hoc test. The significance level was set at *p* value <0.05.

## 3. Results

### 3.1. Extraction Yield

After analyzing the extraction yield of each plant, as shown in Table 1, it was evident that the aqueous extracts of *O. americanum* and *C. filiformis* had higher extraction yields than the hydroethanolic extracts. However, *K. senegalensis* had a higher yield of hydroethanolic extract than aqueous extract. The hydroethanolic extract of *K. senegalensis* had the highest yield, while the hydroethanolic extract of *O. americanum* had the lowest yield.

**Table 1:** Yield (%) at plant extraction.

Yield	*K. senegalensis*	*O. americanum*	*C. filiformis*
Aqueous	7.09%	7.00%	5.20%
Hydroethanolic	16.32%	3.22%	3.14%

### 3.2. Chemical Composition

Based on the analysis of plant powders, it was discovered that different plants have unique profiles of pharmacological groups that exhibit notable biological activities (Table 2). Tannins, specifically gallic and catechin tannins, leucoanthocyanins, and saponins, were present in all five plants studied. However, none of the plants contained anthocyanins, cyanogenic derivatives, O‐heteroside, or C‐heteroside. During the screening, it was observed that *K. senegalensis* and *C. filiformis* have alkaloids and reducing compounds. Coumarins were present exclusively in *C. filiformis*. Quinone derivatives and flavonoids were found in *Khaya senegalensis* and *C. filiformis*. Free anthracenes were only in *K. senegalensis*.

**Table 2:** Secondary metabolites present in plants.

Compounds	*K. senegalensis*	*C. filiformis*	*O. americanum*
Tannin	+	+	+
Catechic tannins	+	+	+
Leuko‐anthocyanins	+	+	+
Gallic tannins	+	+	+
Flavonoids	+	+	—
Anthocyanins	—	—	—
Saponosids	+	+	+
Cyanogenic derivatives	—	—	—
Mucilage	—	—	—
Reducing compounds	+	+	—
Free anthracene	+	—	—
Alkaloids	+	+	—
Quinone derivatives	+	+	—
Coumarins	—	+	—
O‐Hereroside	—	—	—
C‐heteroside	—	—	—

*Note:* “—” absence; “+” presence.

### 3.3. Antimicrobial Activity

#### 3.3.1. Sensitivity of Uropathogenic Strains in the Presence of Aqueous and Hydroethanolic Plant Extracts

Table 3 shows the average size of inhibition diameters for both aqueous and hydroethanolic extracts on various strains. The diameter sizes range from 13.8 to 21.2, depending on the strain and extract used. The hydroethanolic extract of *O. americanum* resulted in the largest diameter of inhibition (20.2 mm) for *Enterobacteriaceae* strains. The minor diameter (13.8 mm) was compared with the aqueous extract of *C. filiformis*. For staphylococci strains, the hydroethanolic extract of *K. senegalensis* showed the largest diameter of inhibition (21.2 mm), while the aqueous extract of *C. filiformis* gave the smallest diameter (16 mm). Furthermore, the uropathogenic strains were not affected by the aqueous extracts of *O. americanum*.

**Table 3:** Inhibition diameter of the extracts on the strains.

	*K. senegalensis*		*O. americanum*		*C. filiformis*		*p* value
	Aqueous	Hydroethanolic	Aqueous	Hydroethanolic	Aqueous	Hydroethanolic	
*Enterobacteriaceae*	15.6 ± 2.5	16.8 ± 0.8	0	20.2 ± 3.5	13.8 ± 1.8	18.6 ± 3.2c	0.003
Staphylococci	16.2 ± 0.8	21.2 ± 1.6	0	19.4 ± 1.8	16.0 ± 0.0	19.2 ± 1.1	0.007

#### 3.3.2. MIC and MBC of Extracts on Uropathogenic Strains

Table 4 presents the values of the extracts’ MIC and MBC for the studied strains. It is worth noting that the MICs range from 3.5 to 7 mg/mL for Enterococci and from 4.5 to 9 mg/mL for Staphylococci. Additionally, the MBC ranges from 16 to 20 mg/mL for Enterococci and 18 to 20 mg/mL for Staphylococci.

**Table 4:** Compilation of the minimum inhibitory concentrations (MIC) and minimum bactericidal concentrations (MBC) values of the extracts on the tested strains.

		*K. senegalensis*		*O. americanum*		*C. filiformis*		*p* value
		Aqueous	Hydroethanolic	Aqueous	Hydroethanolic	Aqueous	Hydroethanolic	
CMI	*Enterobacteriaceae*	4.5 ± 1.12	5.0 ± 0.0	—	3.5 ± 1.4	4.0 ± 1.37	7.0 ± 2.7	0.095
	Staphylococci	5.0 ± 0.0	4.5 ± 1.1	—	4.5 ± 1.1	5.0 ± 0.0	9.0 ± 2.2	0.019
CMB	*Enterobacteriaceae*	16.0 ± 5.5	20.0 ± 0.0	—	16.0 ± 5.5	16.0 ± 5.5	20.0 ± 0.0	0.389
	Staphylococci	20.0 ± 0.0	18.0 ± 4.5	—	20.0 ± 0.0	20.0 ± 0.0	20.0 ± 0.0	0.649
CMI/CMB	*Enterobacteriaceae*	0.3 ± 0.11	0.25 ± 0.0	—	0.25 ± 0.0	0.25 ± 0.0	0.4 ± 0.1	0.085
	Staphylococci	0.25 ± 0.0	0.25 ± 0.0	—	0.25 ± 0.0	0.25 ± 0.0	0.4 ± 0.1	0.003

To determine the extracts’ impact on the studied strains, we calculated the ratio between the CMI and CMB parameters to assess whether they were bactericidal or bacteriostatic. The results showed that the recorded CMB/CMI ratios ranged from 0.25 to 0.45. After comparing the antibacterial activity of the various extract types, we found that the stem plants were more susceptible to hydroethanolic extracts (Table 4).

### 3.4. Anti‐Biofilm Activity of the Plant Extracts Studied

Table 5 presents the results of our evaluation of the anti‐biofilm activity of different plant extracts. The percentage of biofilm inhibition varies across the various extracts. For instance, the hydroethanolic extract of *K. senegalensis* yielded a 41.73% inhibition rate, while the aqueous extract of *O. americanum* achieved 70.27% inhibition in Enterococci. In staphylococcal strains, the percentage inhibition ranges from 40.52% to 74.34%, and the differences are statistically significant (*p* < 0.05). The hydroethanolic extract of *K. senegalensis* achieved the highest inhibition rate of 74.34%.

**Table 5:** Percentage of biofilm inhibition by the extracts.

	Percentage of inhibition of biofilms (%)	
	*Enterobacteriaceae*	Staphylococci
Aqueous *K. senegalensis*	43.34 ± 18.01	65.24 ± 16.49
Hydroethanolic *K. senegalensis*	41.73 ± 18.34	74.34 ± 7.17
Aqueous *O. americanum*	70.27 ± 7.46	44.33 ± 18.23
Hydroethanolic *O. americanum*	55.44 ± 4.20	66.47 ± 5.60
Aqueous *C. filiformis*	48.97 ± 13.60	50.12 ± 16.97
Hydroethanolic *C. filiformis*	55.68 ± 7.41	48.91 ± 14.34
*p* value	0.536	0.004

The extracts inhibited more biofilm‐producing bacteria in staphylococci than in *Enterobacteriaceae*.

### 3.5. Analgesic Activity of the Extracts of the Plants Studied

The results of the analgesic activity of the extracts at 200 mg/kg vary from plant to plant. The percentages of cramp inhibition (PCI) vary from 22.03% (*Khaya senegalensis*) to 67.8% (*C. filiformis*) in the rats treated with the extracts (Table 6). The analgesic activity of *O. Americanum* and *C. filiformis* is comparable with aspirin (*p* > 0.05).

**Table 6:** Effects of extracts on acetic acid‐induced writhing.

	PCI	Std. deviation	Effect/NCe∗	Effect/PC∗∗
*C. filiformis*	67.8%	3.624	0.0001	0.9999
*O. americanum*	47.46%	3.624	0.2923	0.2174
*K. senegalensis*	22.03%	1.812	0.9999	0.0124
Negative control	61.02%	5.436	0.0003	
Positive control	0	0		0.0003

∗Effect or *p* value (< 0.05) shows a significant difference from the negative control (NC).

∗∗Effect or *p* value (< 0.05) expresses a significant difference from the positive control (PT).

### 3.6. Diuretic Activity of Plant Extracts Studied

Total urine volume and urinary electrolyte excretion were measured for extracts, standard (furosemide), and negative control over the 6 h. Compared with control rats, furosemide and hydroethanolic extracts of *K. senegalensis* and *C. filiformis* increased urine output. The hydroethanolic extract of *O. americanum* did not increase urine output in treated rats. No significant difference exists between the volumetric urinary excretion of hydroethanolic extracts of *K. senegalensis* and *C. filiformis* and furosemide (Table 7).

**Table 7:** Effect of extracts on urinary excretion.

	*K. senegalensis*	*O. americanum*	*C. filiformis*	− control	+ control	*p* value
Volumetric urinary excretion	107.80 ± 8.10	17.91 ± 0.80	85.40 ± 6.20	26.70 ± 7.51	88.74 ± 1.00	< 0.0001
Na^+^	179.50 ± 3.96	99.50 ± 2.05	145.40 ± 5.20	104.30 ± 1.20	166.70 ± 4.80	0.001
K^+^	89.95 ± 2.30	37.00 ± 0.85	65.20 ± 2.30	43.00 ± 0.99	78.70 ± 2.40	< 0.0001
Cl^-^	123.45 ± 5.30	61.20 ± 2.71	98.50 ± 5.00	69.19 ± 1.50	119.10 ± 6.80	< 0.0001

The correlation matrix reveals that volumetric urinary excretion is strongly correlated with the presence of ions. In short, when the volumetric urinary excretion increases, Na+, K+, and Cl−excretion increase (Table 8).

**Table 8:** Correlation between volumetric urinary excretion and ions.

	Volumetric urinary excretion	Na^+^	K^+^	Cl^−^
Volumetric urinary excretion	1			
Na^+^	0.978	1		
K^+^	0.967	0.987	1	
Cl^-^	0.968	0.99	0.983	1

### 3.7. Oral Toxicity of Plant Extracts Studied

#### 3.7.1. Effect of Hydroethanolic Plant Extracts on Rat Weight and General Appearance

**Figure 1:**
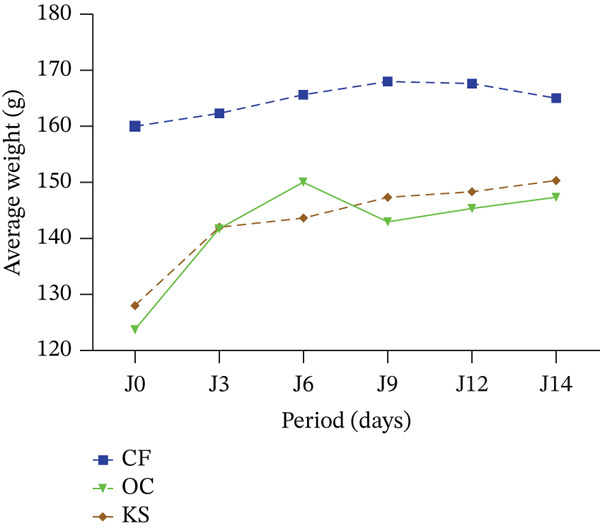
Evolution of the weight of the rats during the test period. CS: *C. filiformis*; OC: *O. americanum*; KS: *K. senegalensis.*

The rats showed no change in general physical appearance and somatomotricity during the observation period. No treatment‐related changes were observed in rats’ stool and urine, eye color, respiration, and behavior over the 14 days following oral administration of a single extract dose at 2000 mg/kg. No death was recorded in the animals during the entire study period. All treated groups showed an increase in body weight throughout the experiment (Figure 1).

#### 3.7.2. Effect of Hydroethanolic Plant Extracts on Rat Weight and General Appearance

Table 9 presents the effect of hydroethanolic extracts of our plants on hematological parameters. No significant difference was noted between the mean levels of the various hematological parameters evaluated in the treated animals.

**Table 9:** Effects of hydroethanolic extracts on hematological parameters of rats.

		*C. filiformis*	*O. americanum*	*K. senegalensis*	ESM	*p* value
Red blood cell number	Day 0	8.35	8.45	8.06	0.29	0.91
	Day 14	8.41	7.48	7.31	0.36	0.07
	Variation	0.05	−0.97	−0.75	0.20	0.03

Hemoglobin level	Day 0	14.53	14.90	14.47	0.47	0.92
	Day 14	15.20	14.03	14.77	0.27	0.05
	Variation	0.67	−0.87	0.30	0.41	0.08

Hematocrit level	Day 0	46	47.33	46	1.06	0.83
	Day 14	54.66	51.00	51.67	1.80	0.08
	variation	8.66	3.66	5.67	1.17	0.04

Mean globular volume	Day 0	55.33	56.33	57.33	1.13	0.69
	Day 14	65.00	68.67	71.00	1.39	0.10
	Variation	9.66	12.33	13.67	0.62	0.02

Mean corpuscular hemoglobin content	Day 0	17.33	17.67	18	0.27	0.45
	Day 14	18.33	19.00	20.67	0.70	0.26
	Variation	1.00	1.33	2.67	0.64	0.59

Mean corpuscular hemoglobin concentration	Day 0	31.33	31.33	31.33	0.44	0.93
	Day 14	27.66	27.33	28.67	0.58	0.73
	Variation	−3.66	−4.00	−2.67	0.86	0.97

White blood cells	Day 0	7.07	8.12	8.63	0.81	0.60
	Day 14	8.12	7.72	7.92	0.98	0.96
	Variation	1.02	−0.40	−0.71	1.01	0.87

Neutrophil	Day 0	10.00	13.33	9.00	5.88	0.96
	Day 14	10.33	18.67	15.33	4.11	0.57
	Variation	0.33	5.33	6.33	6.55	0.92

Eosinophil	Day 0	0.67	0.00	0.67	0.67	0.75
	Day 14	1.00	1.67	0.67	0.38	0.47
	Variation	0.33	1.67	0.00	0.83	0.42

Monocytes	Day 0	15.00	26.00	18.67	5.95	0.81
	Day 14	14.00	11.67	13.33	3.03	0.87
	Variation	−1.00	−14.33	−5.30	6.36	0.80

Lymphocyte	Day 0	74.67	60.67	71.67	2.36	0.01
	Day 14	75.00	67.33	68.33	1.93	0.14
	Variation	0.33	6.67	−3.33	3.12	0.25

Platelets	Day 0	716.00	772.67	946.00	67.75	0.78
	D14	741.00	738.67	840.33	61.24	0.56
	Variation	25.00	−34.00	−105.67	50.19	0.35

Table 10 shows the extracts’ effect on the biochemical parameters. No significant difference was reported between the mean levels of the different biochemical parameters evaluated in the treated animals.

**Table 10:** Effects of hydroethanolic extracts on the biochemical parameters of rats.

		*C. filiformis*	*O. americanum*	*K. senegalensis*	ESM	*p* value
Glycemia	Day 0	0.48	0.44	0.32	0.03	0.06
	Day 14	0.39	0.38	0.27	0.05	0.29
	Variation	−0.08	−0.05	−0.05	0.03	0.70

Urea	Day 0	0.56	0.70	0.49	0.06	0.46
	Day 14	0.42	0.53	0.43	0.04	0.47
	Variation	−0.14	−0.17	−0.06	0.04	0.27

Creatine	Day 0	6.27	6.20	5.43	0.50	0.15
	Day 14	7.35	5.65	6.59	0.30	0.02
	Variation	1.07	−0.55	1.16	0.39	0.03

Total cholesterol	Day 0	1.20	1.16	1.22	0.04	0.87
	Day 14	1.44	1.15	1.18	0.15	0.69
	Variation	0.24	−0.01	−0.04	0.14	0.74

High‐density lipoprotein cholesterol	Day 0	0.52	0.42	0.45	0.04	0.59
	Day 14	0.49	0.44	0.52	0.04	0.54
	Variation	−0.03	0.02	0.07	0.04	0.34

Triglycerides	Day 0	0.85	0.50	0.66	0.08	0.16
	Day 14	0.64	0.56	0.93	0.08	0.03
	Variation	−0.21	0.05	0.27	0.08	0.11

Aspartate aminotransferase	Day 0	23.66	23.66	21.00	2.52	0.44
	Day 14	55.00	17.66	24.33	6.98	0.14
	Variation	31.33	−6.00	3.33	5.32	0.07

Alanine aminotransferase	Day 0	26.66	22.33	18.66	3.96	0.65
	Day 14	49.33	36.66	32.00	3.60	0.13
	Variation	22.66	14.33	13.33	2.61	0.26

#### 3.7.3. Result of the Histopathological Study

**Figure 2:**
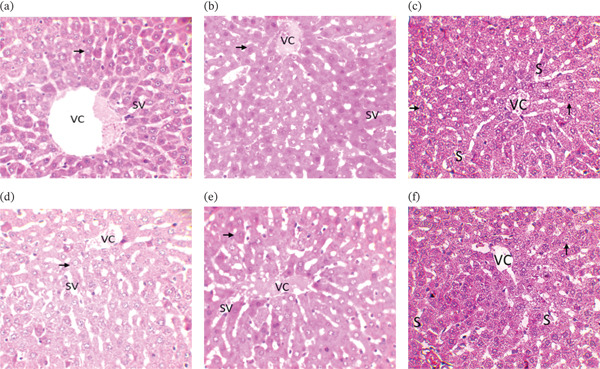
Liver histology in acute oral toxicity test of KS extract at 400× magnification. Liver histology at acute oral toxicity test of tested extract at 400× magnification. (a) Liver histology control batch of treatments with *K. senegalensis* extract; (b) liver histology control batch of treatments with *O. americanum* extract, (c) liver histology control batch of treatments with *C. filiformis* extract, (d) liver histology in the acute oral toxicity test using the *K. senegalensis* extract, (e) liver histology in the acute oral toxicity test using the extract of *O. americanum*, and (f) liver histology in the acute oral toxicity test using the extract of *C. filiformis*.

**Figure 3:**
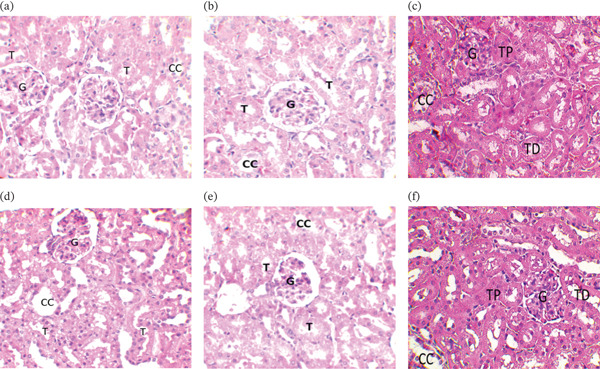
Kidney histology at acute oral toxicity test of tested extract at 400X magnification. (a) Renal histology control batch of treatments with *K. senegalensis* extract; (b) Renal histology control batch of treatments with *O. americanum* extract, (c) Renal histology control batch of treatments with *C. filiformis* extract, (d) Renal histology in the acute oral toxicity test using the *K. senegalensis* extract, (e) Renal histology in the acute oral toxicity test using the extract of *O. americanum*, (f) renal histology in the acute oral toxicity test using the extract of *C. filiformis*.

Figures 2 and 3 show the results of histopathological examinations of the kidney and liver of rats treated with extracts of *K. senegalensis*, *O. americanum*, and *C. filiformis*. In the acute oral toxicity test, the livers of rats force‐fed with the extracts showed no visible atypia. Normal‐looking hepatocytes (arrows) are arranged in radial cords around the central vein (VC). Venous sinusoids (S) are visible, as seen in control rats. The renal parenchyma of the rats force‐fed with the extracts also kept its typical appearance, as observed in the control rats. Glomeruli (G), proximal and distal tubules (T), and collecting ducts (CC) showed no visible atypia.

## 4. Discussion

Extracting active substances from medicinal plants has been a reliable and essential method for developing new therapeutic remedies. In our study, we used hydroethanolic and aqueous extractions to calculate the yields of various plants. The best extraction yield was obtained with aqueous extracts from *O. americanum* and *C. filiformis*. This indicates that the constituents of these plants pass more easily in water than in the hydroethanolic solution, which is in line with traditional use. However, for *K. senegalensis*, the best yield is obtained with ethanol. The type of extraction solvent closely affects the yield [26]. When comparing our results with existing literature [27–30], we found variations in extraction yields. This could be due to various factors such as the geographical origin of the plant, the pedoclimatic conditions, the age of the plant, the treated part, the freshness of the plant material, the harvest period, the environment, and the method of extraction [31].

Plants are a great source of phytochemicals that can be beneficial to humans. Our plants were analyzed for bioactive molecules through qualitative phytochemical screening, which revealed the presence of various families of molecules such as tannins, alkaloids, Leuco‐anthocyanins, reducing compounds, coumarins, quinone derivatives, and flavonoids. Research on *K. senegalensis*, *O. americanum*, and *C. filiformis*, has confirmed the existence of these molecules in these plants [27, 29, 32, 33]. Mucilage was not found in our tested plants. However, some authors reported the presence of mucilage in *K. senegalensis* [34], *O. americanum* [28], and *C. filiformis* [29]. The observed differences could be due to differences in study methods, harvest locations, and the organs used [35]. The three tested plants do not contain harmful substances such as cyanogenic derivatives, anthracenes, O‐heterosides, and C‐heterosides. The absence of cyanogenic derivatives is reassuring for consumers as they can poison the body [36]. These plants are rich in secondary metabolites with various biological and physiological benefits such as antibacterial, antiviral, anticancer, antitumor, antifungal, analgesic, anti‐inflammatory, gastrointestinal, diuretic, and antioxidant properties [37, 38]. This diversity of secondary metabolites can help manage UTIs and explains why they are used in traditional medicine. It is important to note that the effectiveness of plant extracts depends on the concentration of active ingredients [39], so isolating these active ingredients is necessary.

Our research delved into the antimicrobial effects of various plants by evaluating their antibacterial and anti‐biofilm properties. We observed that the plants’ antibacterial activity varied depending on the bacterial strain and the type of extract employed. Thus, *K. senegalensis*, *O. americanum*, and *C. filiformis* extracts were effective against uropathogenic strains, aligning with previous studies that have highlighted their antibacterial activity [40–42]. Our study also established that secondary metabolites, such as phenolic compounds, tannins, alkaloids, flavonoids, and saponosides, have antimicrobial properties that correlate with antibacterial activity [43–45]. We compared the antimicrobial activities of the different plants and discovered that *K. senegalensis* had the most potent antibacterial effect. Furthermore, we observed that the type of solvent used during extraction significantly impacted the plants’ antibacterial effectiveness, with hydroethanolic extracts inhibiting bacterial strains more effectively than aqueous extracts due to the higher affinity of plant substances with ethanol during extraction [46]. Our study focused on uropathogenic strains that are multi‐resistant. While our results indicated that the active substances in the studied plants had a bactericidal effect, further research is needed to understand these metabolites’ modes of action. The CMB/CMI ratio values ranged from 0.25 to 0.45, further indicating the bactericidal effect of the bioactive compounds in the extracts. This effect may manifest in bacteria through the destruction of microbial walls or the inactivation of enzymes.

Preventing or inhibiting biofilm formation is crucial in managing infections, as biofilms provide bacteria with better protection against host defenses and antibiotics. In our study, we found that extracts from the plants we examined had inhibitory effects on the ability of uropathogenic bacteria to form biofilms at varying percentages. Medicinal plants are an important source of anti‐biofilm substances due to their richness in biologically active compounds, according to Carette et al. [10]. Natural products’ anti‐biofilm properties are primarily based on specific aspects, such as suppressing cell adhesion and attachment, inhibiting polymer matrix formation, interrupting extracellular matrix generation, and reducing virulence factors’ production, as noted by Lu et al. [47]. According to Ghosh et al. [48], organic compounds in plant extracts can alter surface characteristics (roughness, texture, hydrophobicity, and hydrophilicity), preventing bacterial adhesion and biofilm formation. Thus, the anti‐biofilm effects of the studied plant materials may be attributed to their metabolites’ capabilities. However, the molecules or bioactive components responsible for our plants’ anti‐biofilm effects remain unknown and require further study.

Our research findings indicate that staphylococci are more receptive to plant‐based antimicrobials than *Enterobacteriaceae*. Previous studies have shown that Gram‐positive bacteria, specifically *S. aureus*, are generally more responsive to antibiotics than Gram‐negative bacteria [49]. The structure of Gram‐negative bacteria, with an inner and outer membrane, makes it difficult for drugs and antibiotics to penetrate the cell [50]. In contrast, Gram‐positive bacteria lack this barrier, making them more vulnerable to antimicrobial agents. The active components in plant extracts likely had to overcome this permeability barrier to access their targets within the bacterial cell.

Plants have been widely used in traditional pain treatment worldwide. Analgesic activity has been observed in the hydroethanolic extracts, with *K. senegalensis* showing weak activity. Other experiments have shown significant analgesic activity in extracts of *C. filiformis* [51] and *O. americanum* [52]. Our phytochemical study has identified numerous constituents that may be responsible for this activity. Based on these findings, we recommend using the aqueous extract of these two recipes to alleviate peripheral pain associated with UTIs.

When treating UTIs, preparations with diuretic effects are often used in addition to antibiotics. Our study examined the diuretic activity of hydroethanolic extracts at a dosage of 200 mg/kg and compared their effects to those of furosemide, a commonly used loop diuretic in clinical practice. We also evaluated the extracts’ impact on electrolyte balance. *K. senegalensis* and *C. filiformis* showed significant increases in the urinary excretion of Na+, K+, and Cl− ions, indicating diuretic activity. Previous studies by Sakshy et al. [53] have also confirmed the diuretic activity of *C. filiformis*, due to secondary metabolites such as flavonoids and saponins. Our preliminary phytochemical investigation revealed the presence of these metabolites in our plants, suggesting they may be responsible for the diuretic activity. Alkaloids and other metabolites may also be present. Further research is needed to determine the exact constituents accountable for the extracts’ diuretic activity and better understand their mechanism of action on the kidney and urine volume.

It is essential to assess the toxicity of herbal medicinal preparations or drugs, as they can adversely affect humans. Our study conducted the acute toxicity test to evaluate the adverse effects shortly after administering a single dose of 2000 mg/kg of a test substance [54]. Animals treated with our extracts did not exhibit any signs of physical illness or death throughout the study, and their body weight gradually increased as expected. This suggests that our plants do not have any adverse effects on the predicted growth of rats. Assessing blood parameters is crucial in determining toxicity risks, as changes in hematological and biochemical parameters can indicate toxicity [54]. In our study, all hematological changes observed were within normal limits, indicating that the physiological mechanisms reflected by these parameters, such as respiration, immunology, and anemia, were not altered in rats treated with high doses [55]. The analysis of biochemical parameters of the liver, kidneys, and lipids also showed minor fluctuations, but no membrane‐stabilizing effect was observed on these organs [56]. High levels of urea and creatinine in the serum indicate kidney tissue damage, while high cholesterol can contribute to cardiovascular disease. However, our histopathological examinations did not reveal any significant alterations in the vital organs of rats treated with our extracts [48]. Comparing our data with those from other studies, we observed variable relationships. Some studies have found that certain extracts can cause toxicity in biochemical, hematological, and histopathological parameters when administered chronically for several weeks [34, 57]. However, other studies have shown that regular therapeutic doses of certain extracts do not produce severe toxicological effects on rats [58–60].

## 5. Conclusion

This study has revealed a range of biological activities, such as antibacterial, anti‐biofilm, analgesic, and diuretic effects, in addition to the non‐toxicity of the aqueous and hydroethanolic extracts from five medicinal plants. The active ingredients in the extracts are believed to be responsible for the observed pharmacological activities. These findings support the traditional use of these plants for treating UTIs in Benin. Further research is needed to identify and isolate the active compounds responsible for these properties and to comprehend their mechanisms of action at the molecular and tissue levels. These investigations could lead to improved traditional medicine that combines the benefits of these plants.

## Author Contributions

Conceptualization: Funkè F. Assouma and Haziz Sina; data curation, Funkè F. Assouma, Machioud Maxime Sangaré, Atchadé Pascal Tchogou, Gautier Roko, Bawa Boya, Basile Konmy, and Haziz Sina; formal analysis: Funkè F. Assouma, Bassitath Bello, Rachidatou Karimou, and Durand Dah‐Nouvlessounon; funding acquisition: Adolphe Adjanohoun, Lamine Baba‐Moussa, and Haziz Sina; investigation: Funkè F. Assouma, Cyrille A. Vodounon, and Bawa Boya; methodology: Funkè F. Assouma, Machioud Maxime Sangaré, Bawa Boya, Atchadé Pascal Tchogou, Rachidatou Karimou, and Haziz Sina; resources: Lamine Baba‐Moussa and Haziz Sina; supervision: Adolphe Adjanohoun, Lamine Baba‐Moussa, and Haziz Sina; validation: Gautier Roko, Machioud Maxime Sangaré, and Atchadé Pascal Tchogou; writing original draft, Funkè F. Assouma and Rachidatou Karimou; writing review and editing: Durand Dah‐Nouvlessounon, Cyrille A. Vodounon, Adolphe Adjanohoun, Lamine Baba‐Moussa, and Haziz Sina.

## Funding

No funding was received for this manuscript.

## Disclosure

All authors have read and agreed to the published version of the manuscript.

## Ethics Statement

The experimental protocol (approval no. UAC/EDSVT/11614210), involving the use of Wistar rats for acute toxicity studies, was reviewed and approved by the Ethics Committee of the Doctoral School of Life and Earth Sciences (EDSVT) of the University of Abomey‐Calavi (UAC). All animal procedures were conducted in accordance with institutional ethical guidelines and complied with the ARRIVE guidelines for the reporting of animal research.

## Consent

The authors have nothing to report.

## Conflicts of Interest

The authors declare no conflicts of interest.

## Data Availability

The data used to support this work’s findings are available from the corresponding author upon request.
